# Retinal Vasculopathy With Cerebral Leukoencephalopathy Mimicking a Brain Tumor: A Case Report

**DOI:** 10.7759/cureus.104149

**Published:** 2026-02-23

**Authors:** Juan Carlos Villegas Hernández, Christian Edward Sánchez Sánchez, Karla Karyme Sahagún Leyva, Brenda Selene Lozano Santos, Alma Ortiz-Plata, Nancy Monroy-Jaramillo, Martha Lilia Tena Suck

**Affiliations:** 1 Neuropathology, Instituto Nacional de Neurología y Neurocirugía, Mexico City, MEX; 2 Neuroradiology, Instituto Nacional de Neurología y Neurocirugía, Mexico City, MEX; 3 Pathology, Hospital Metropolitano Dr. Bernardo Sepúlveda, San Nicolás de los Garza, MEX; 4 Pathology, Centro Médico Nacional "Manuel Ávila Camacho" IMSS, Puebla de Zaragoza, MEX; 5 Laboratory of Experimental Neuropathology, Instituto Nacional de Neurología y Neurocirugía, Mexico City, MEX; 6 Neurogenetics, Instituto Nacional de Neurología y Neurocirugía, Mexico City, MEX

**Keywords:** cerebral leukoencephalopathy, demyelination, genetic vasculopathy, pseudotumoral brain lesion, retinal vasculopathy with cerebral leukoencephalopathy, rim-enhancing lesion, trex1 mutation

## Abstract

Retinal vasculopathy with cerebral leukodystrophy (RVCL) is an adult-onset, autosomal dominant disorder caused by heterozygous C-terminal frameshift mutations in *TREX1*, leading to mislocalization of its normally perinuclear exonuclease. Patients typically present with progressive visual impairment and neurological decline, while brain magnetic resonance imaging (MRI) commonly demonstrates punctate white matter lesions or tumor-like rim-enhancing masses. We report the case of a 53-year-old woman who initially presented with progressive headaches, followed by visual loss, cognitive impairment, and focal neurological deficits. Brain MRI revealed a left frontal white matter rim-enhancing lesion highly suggestive of a high-grade glioma. Histopathological evaluation demonstrated ischemic white matter injury, marked vascular hyalinization with fibrinoid necrosis, and prominent dystrophic calcifications. Ultrastructural analysis revealed multilaminated basement membranes and granular osmophilic deposits. Genetic testing, which had been performed previously, later demonstrated a pathogenic *TREX1* mutation, establishing the diagnosis of RVCL. This case highlights the importance of considering RVCL in the differential diagnosis of tumor-like white matter lesions and underscores the clinical value of detailed histopathological and ultrastructural characterization, which is seldom available in reported cases.

## Introduction

Retinal vasculopathy with cerebral leukoencephalopathy (RVCL) is a rare autosomal dominant small-vessel vasculopathy caused by heterozygous C-terminal frameshift mutations in the *TREX1 *gene [1]. *TREX1* encodes a 3′-5′ DNA exonuclease involved in the clearance of cytosolic nucleic acids; pathogenic truncating mutations result in mislocalization of the protein and endothelial dysfunction rather than a primary inflammatory process [1].

RVCL-S encompasses previously described entities that were initially reported as distinct disorders, including cerebroretinal vasculopathy (CRV), hereditary vascular retinopathy (HVR), and hereditary endotheliopathy with retinopathy, nephropathy, and stroke (HERNS), which are now recognized as part of the same genetic spectrum [1,2].

Five recurrent C-terminal frameshift mutations have been identified (V235fs, T236fs, T249fs, R284fs, and L287fs), all inherited in an autosomal dominant pattern, and fewer than 200 affected individuals have been reported worldwide, underscoring the rarity of this condition [1-3].

Neurological involvement occurs in approximately 80% of patients and includes focal neurological deficits, migraine, cognitive impairment, psychiatric symptoms, and seizures. Neuroimaging typically demonstrates punctate white matter hyperintensities, nodular or rim-enhancing mass-like lesions, and intracranial calcifications; these findings mimic high-grade gliomas or tumefactive demyelinating lesions and could lead to biopsy [3,4].

Systemic manifestations are present in most patients and include liver disease, anemia, nephropathy, hypertension, Raynaud phenomenon, and gastrointestinal bleeding, reflecting the underlying systemic microangiopathy [2,3].

Pathologically, RVCL is characterized by a non-inflammatory small-vessel vasculopathy with vascular wall thickening, luminal narrowing, and secondary ischemic white matter injury. Because brain biopsy is rarely performed, detailed histopathological and ultrastructural descriptions of these tumor-like lesions remain limited in the literature.

We report a familial case of RVCL in a 53-year-old woman presenting with a rim-enhancing tumor-like white matter lesion initially suspected to represent a high-grade glioma, highlighting the clinicopathologic correlation and the diagnostic importance of recognizing this entity.

## Case presentation

A 53-year-old woman with a family history of a leukodystrophy-like disorder affecting her mother and two siblings, as well as two children with neurodevelopmental delay of suspected but unconfirmed leukodystrophy, was evaluated for progressive neurological symptoms. Her gynecologic history included menarche at 12 years of age, menopause at 39 years, three pregnancies, two live births, and one spontaneous abortion.

In May 2024, she developed progressive bifrontal oppressive headaches, predominantly in the morning, with a maximum intensity of 10/10 on the visual analog scale. The headaches significantly limited daily activities and partially improved with rest. Over the following months, she developed progressive right-sided weakness, and in September 2024, dysarthria became evident.

**Figure 1:**
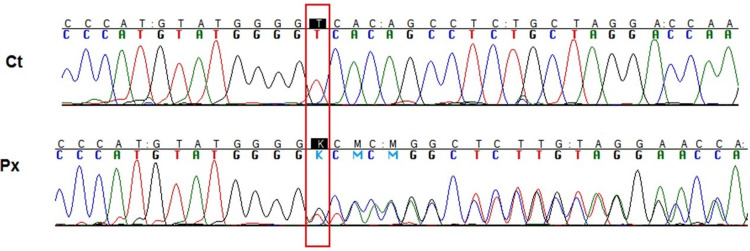
TREX1 gene sequencing analysis Electropherogram showing a heterozygous guanine insertion in the coding exon of *TREX1* (c.3688_3689insG; p.Val235Glyfs*240), highlighted by a red box. The upper panel corresponds to the partial sequence of a healthy control, while the lower panel shows the partial sequence of the affected patient with RVCL. This variant results in a frameshift and premature truncation of the protein. RVCL: Retinal vasculopathy with cerebral leukodystrophy.

Given the progressive neurological deterioration and the strong family history suggestive of an inherited small-vessel disorder, molecular genetic testing was requested. Sequence analysis identified a heterozygous frameshift mutation in the *TREX1 *gene (c.3688_3689insG; p.Val235Glyfs*240), a known pathogenic variant associated with retinal vasculopathy with cerebral leukoencephalopathy. The insertion of a single guanine nucleotide resulted in premature truncation of the *TREX1 *protein, as demonstrated by Sanger sequencing (Figure 1).

**Figure 2:**
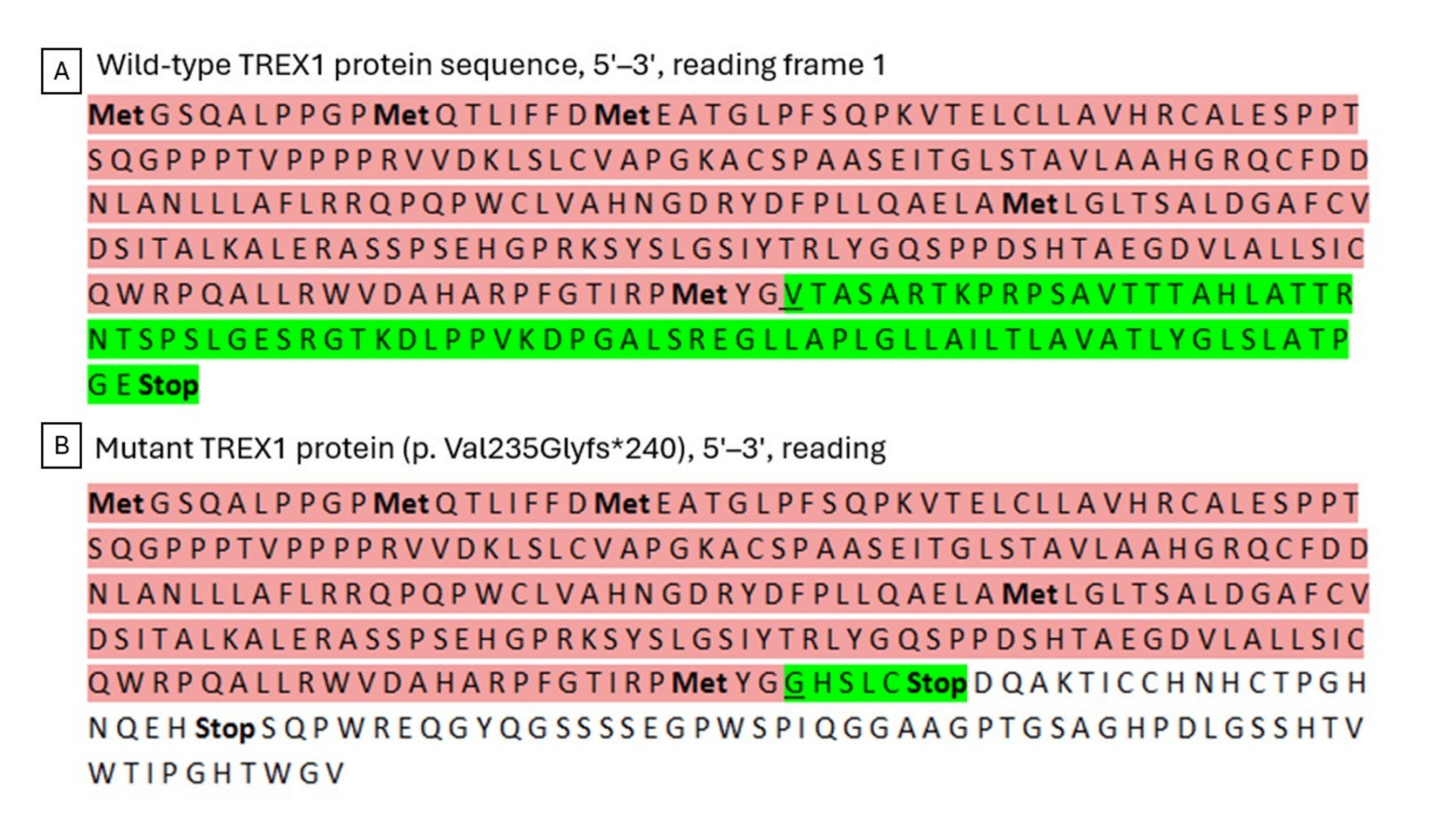
TREX1 exonuclease protein sequence Schematic representation of the wild-type *TREX1* protein (314 amino acids, A) compared with the truncated mutant *TREX1 *protein (239 amino acids, B). The conserved N-terminal region preceding the mutation site is shown in pink. The altered C-terminal region resulting from the frameshift mutation p.Val235Glyfs*240 is shown in green, illustrating the loss of the terminal portion of the protein.

The predicted structural consequence of this mutation, leading to loss of the C-terminal region of the exonuclease, is shown by comparison between the wild-type and mutant proteins (Figure 2).

By April 2025, the patient reported new difficulty performing mathematical operations. On neurological examination, she was awake and fully alert. Speech was dysarthric, with impaired articulation of certain words, while language comprehension and spontaneous expression were preserved. Judgment remained intact. Calculation was impaired, as evidenced by difficulty performing simple additions and subtractions, whereas abstract thinking was preserved.

Cranial nerve examination showed normal visual acuity in the right eye (20/20) and decreased visual acuity in the left eye (20/100), improving to 20/70 with pinhole correction. Color vision assessed with Ishihara plates was normal in the right eye (8/8) and absent in the left eye (0/8). Visual field testing demonstrated a central scotoma in the left eye, more pronounced in the inferior and nasal quadrants. Fundoscopic examination was unremarkable. Extraocular movements were full, with intact primary gaze and smooth pursuit. A mild right central facial palsy was observed.

**Figure 3:**
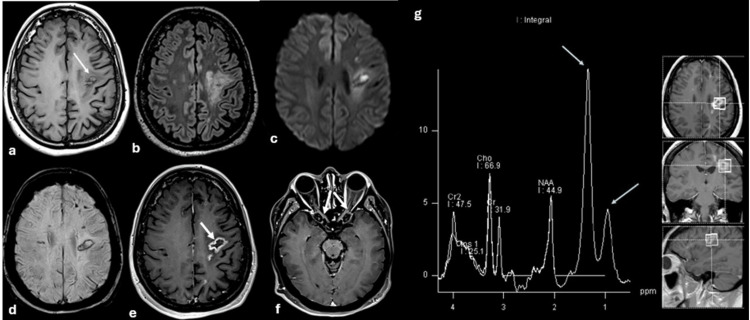
Brain magnetic resonance imaging findings Brain magnetic resonance imaging (MRI), including non-contrast and contrast-enhanced sequences. Axial images of the lesion are shown in T1-weighted, fluid-attenuated inversion recovery (FLAIR), diffusion-weighted imaging (DWI), susceptibility-weighted imaging (SWI), post-contrast T1-weighted (T1+Gd), and magnetic resonance spectroscopy (A–G). (a) T1-weighted image demonstrates a heterogeneous pseudotumoral lesion in the left frontal lobe with a hypointense peripheral rim and a hyperintense central component (thin white arrow). (b) FLAIR image shows involvement of the perilesional white matter without significant mass effect. (c) DWI demonstrates diffusion restriction involving both the lesion and the adjacent perilesional white matter. (d) SWI reveals a paramagnetic rim consistent with hemorrhagic components, as well as multiple smaller multifocal lesions. (e) Post-contrast T1-weighted image shows ring enhancement of the lesion. (f) Additional contrast enhancement is observed along both optic nerves (thick white arrows). (g) Magnetic resonance spectroscopy (TE 35 ms) demonstrates decreased N-acetylaspartate (NAA) without primary or secondary choline peaks or lipid/lactate peaks (arrows).

Brain magnetic resonance imaging (MRI) revealed a ring-enhancing lesion involving the left frontal lobe, highly suspicious for a high-grade glioma (Figure 3).

Given the progressive clinical course and radiologic findings, surgical intervention was undertaken, and the lesion was resected via neuronavigation-guided craniotomy.

**Figure 4:**
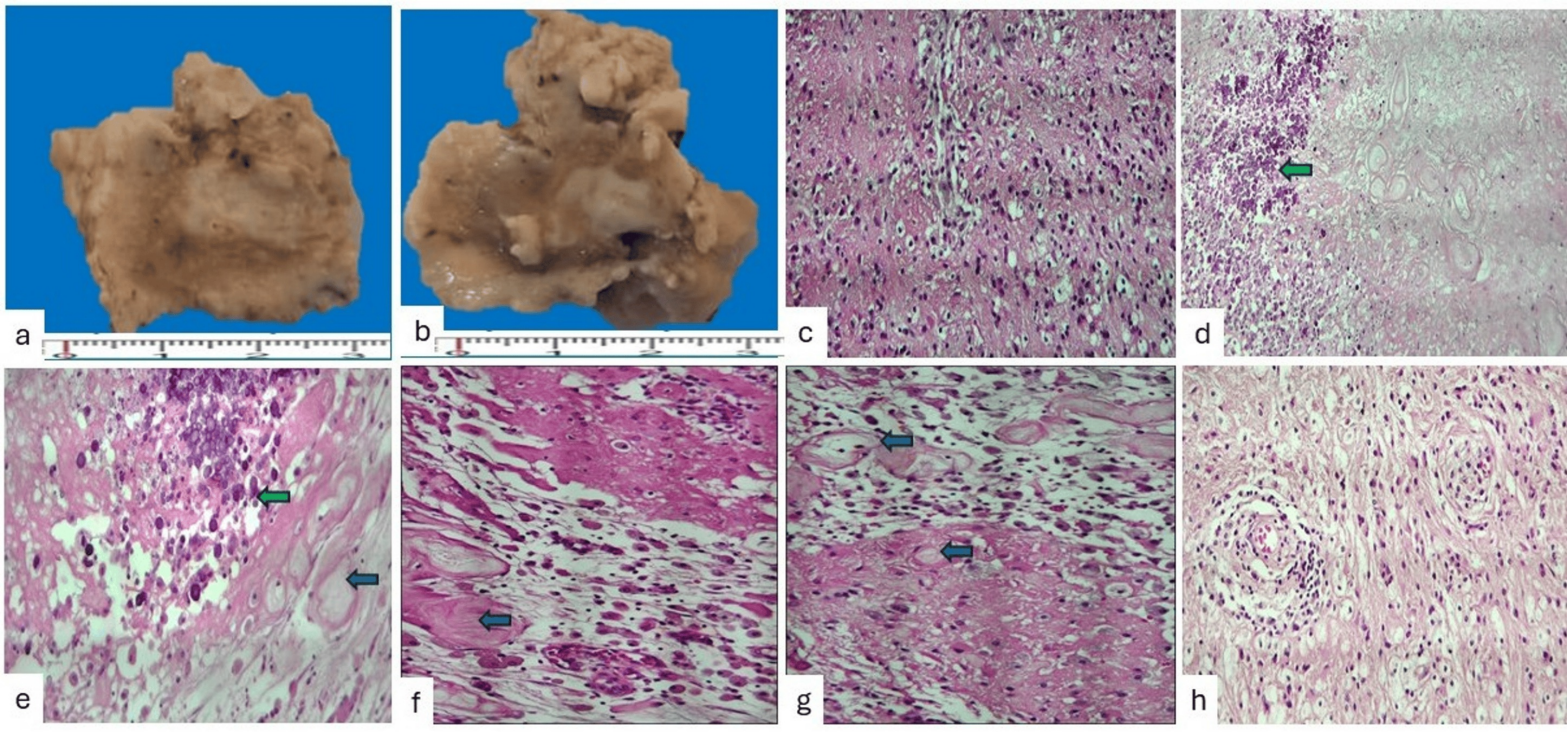
Gross and histopathological findings of the brain lesion (a, b) Gross examination of the resected brain tissue showing a yellowish fragment measuring 25 × 20 × 20 mm. On cut surface, cerebral cortex and white matter are identified, with the white matter displaying firm, whitish fibrous areas alternating with softened necrotic regions. (c) Cerebral cortex showing neuronal loss, ischemic neuronal changes, and abundant foamy macrophages (H&E) (100X). (d) White matter with marked hyalinization of small vessels and dystrophic calcifications (H&E) (40X). (e) Higher magnification highlighting areas of fibrinoid necrosis with prominent calcifications (H&E) (100X). (f, g) Edematous white matter with increased numbers of foamy macrophages (H&E) (40X). (h) Small blood vessels showing perivascular infiltration by mature lymphocytes within the Virchow–Robin space (H&E) (40X).

Gross examination of the surgical specimen revealed a yellow-white brain tissue fragment measuring 25 × 20 × 20 mm, including cortex and underlying white matter. The white matter demonstrated alternating firm, fibrous areas and softened regions with a necrotic appearance (Figure 4, Panels a and b). Histological examination showed cortical neuronal loss with ischemic changes and abundant foamy macrophages (Figure 4, Panel c). The white matter exhibited marked vascular hyalinization with dystrophic calcifications (Figure 4, Panel d). At higher magnification, areas of fibrinoid necrosis and prominent calcifications were identified (Figure 4, Panel e). White matter edema with foamy macrophages was also observed (Figure 4, Panels f and g). There is mature lymphocytic infiltration of the Virchow-Robin spaces (Figure 4, Panel h).

**Figure 5:**
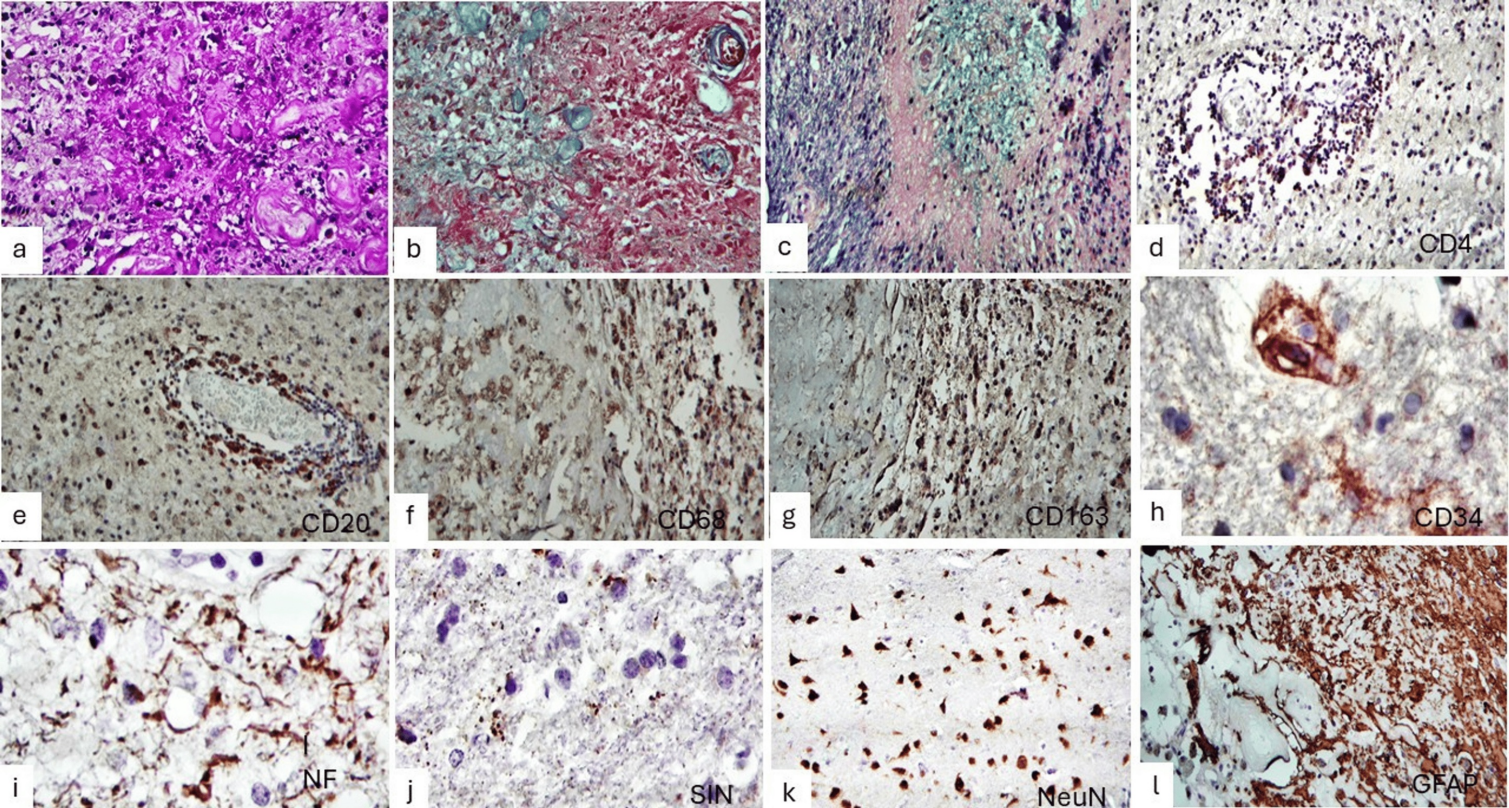
Histochemical and immunohistochemical features (a) PAS stain demonstrating PAS-positive macrophages and thickened vessel walls (40X). (b) Masson trichrome stain highlighting hyalinized vessels in blue (40X). (c) Klüver–Barrera stain showing focal areas of demyelination (40X). (d) Focal perivascular CD4-positive lymphocytic infiltrate. (e) Diffuse CD20-positive immunoreactivity in perivascular inflammatory cells. (f, g) CD68- and CD163-positive macrophages in areas of tissue injury. (h) CD34 immunostaining highlighting endothelial cells with elongated processes. (i) Neurofilament immunostaining showing axonal fragmentation and thickening. (j) Synaptophysin immunostaining with occasional tortuous granular deposits. (k) NeuN immunostaining highlighting retractile neurons. (l) GFAP immunostaining demonstrating densely cellular gliosis as well as depopulated areas with prominent perivascular astrocytic gliosis. (Immunohistochemistry magnifications: CD4, CD20, CD68, and CD163, 40X; CD34, NF, SIN, GFAP, and NeuN, 400X.) PAS: Periodic acid Schiff; GFAP: Glial fibrillary acidic protein.

Histochemical staining demonstrated PAS-positive macrophages and thickened vessel walls (Figure 5, Panel a), hyalinized vessels highlighted by Masson trichrome staining (Figure 5, Panel b), and areas of demyelination on Klüver-Barrera staining (Figure 5, Panel c). Immunohistochemical analysis revealed focal perivascular CD4-positive lymphocytes (Figure 5, Panel d) and diffuse CD20-positive immunoreactivity (Figure 5, Panel e). CD68 and CD163 staining highlighted numerous macrophages and microglial cells (Figure 5, Panels f and g). CD34 immunostaining labeled endothelial cells with elongated and irregular processes (Figure 5, Panel h). Neurofilament staining demonstrated axonal fragmentation and thickening (Figure 5, Panel i), while synaptophysin showed occasional tortuous granular deposits (Figure 5, Panel j). NeuN highlighted residual retractile neurons (Figure 5, Panel k), and glial fibrillary acidic protein (GFAP) staining demonstrated areas of dense gliosis as well as hypocellular regions with prominent perivascular gliosis (Figure 5, Panel l).

Based on the integration of clinical findings, neuroimaging, histopathology, and genetic testing, a final diagnosis of retinal vasculopathy with cerebral leukoencephalopathy associated with a pathogenic *TREX1* mutation was established. Ultrastructural analysis was performed as a complementary study to further characterize the microvascular alterations in this rare biopsied lesion.

**Figure 6:**
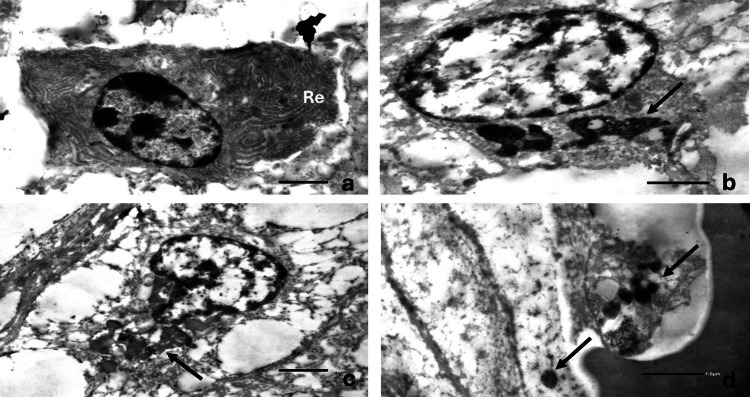
Ultrastructural findings on transmission electron microscopy (a) Cells showing abundant concentric arrays of dilated endoplasmic reticulum (Re) (×4,000; scale bar = 2 µm). (b) Cells containing clusters of electron-dense cytoplasmic material (arrow) (×4,000; scale bar = 2 µm). (c) Endothelial cell with electron-dense material within the cytoplasm (arrow) (×4,000; scale bar = 2 µm). (d) Small vessels showing granular osmophilic deposits (arrows) (×10,000; scale bar = 1 µm). Transmission electron microscopy micrographs stained with uranyl acetate and lead citrate.

Electron microscopy of tissue retrieved from paraffin-embedded blocks demonstrated cells with abundant concentric rough endoplasmic reticulum (Figure 6, Panel a), some containing electron-dense cytoplasmic material (Figure 6, Panel b). Endothelial cells showed granular osmophilic deposits and dense intracytoplasmic inclusions (Figure 6, Panels c and d), supporting the presence of chronic small-vessel injury.

After establishment of the histopathological and genetic diagnosis, oncologic treatment was not initiated. Given the absence of disease-modifying therapy for RVCL, the patient was managed with supportive measures and rehabilitation. Short-term follow-up showed clinical stability without new neurological deficits.

## Discussion

RVCL is caused by mutations in *TREX1 *that result in mislocalization of the exonuclease and dysregulated innate immune signaling. *TREX1 *dysfunction results in cytosolic DNA accumulation and activation of the cGAS-STING pathway with subsequent type I interferon signaling [1,3,5]. Brain involvement occurs in the majority of patients, frequently manifesting as focal deficits, migraine, cognitive decline, and psychiatric symptoms [3]. Neuroimaging typically shows punctate white matter lesions and tumefactive rim-enhancing masses that may mimic high-grade glioma or inflammatory demyelinating disease [4,6].

In the present case, the patient developed a progressive focal neurological syndrome with a rim-enhancing frontal white matter lesion highly suggestive of a neoplastic process. This radiological pattern has been described in RVCL and represents one of the most challenging diagnostic scenarios, often leading to biopsy or surgical resection [4,6]. The absence of significant mass effect and the presence of multifocal susceptibility changes may provide clues to a vascular etiology, although these findings are not specific.

Histopathological examination demonstrated ischemic white matter injury, marked vascular hyalinization with fibrinoid necrosis, dystrophic calcifications, and abundant foamy macrophages, consistent with a non-inflammatory small-vessel vasculopathy. These findings align with previously described neuropathological features of RVCL, including thickened vessel walls, luminal narrowing, and secondary demyelination related to chronic ischemia [2,7]. The perivascular lymphocytic infiltrates observed in our case were mild and are best interpreted as reactive rather than indicative of primary vasculitis.

The differential diagnosis of a tumefactive white matter lesion with vascular changes is broad and includes inherited and acquired small-vessel diseases. Leukodystrophies and genetic leukoencephalopathies may present with progressive white matter abnormalities; however, they typically lack the severe small-vessel hyalinization and fibrinoid necrosis seen in RVCL [8,9]. CADASIL, caused by NOTCH3 mutations, is characterized by granular osmiophilic material deposition in vessel walls and subcortical infarcts but usually lacks prominent calcifications and fibrinoid necrosis [10-12]. Similarly, COL4A1-related disorders produce a hereditary small-vessel angiopathy with hemorrhagic manifestations and basement membrane defects but show a different clinicoradiologic spectrum and earlier onset in many cases [13-15]. Systemic lupus erythematosus may also produce small-vessel vasculopathy and white matter lesions; however, the absence of systemic autoimmune features and the presence of a pathogenic *TREX1 *mutation favored RVCL in this patient [16].

An important aspect of this case is the availability of detailed histopathological and ultrastructural analysis, which is rarely reported because biopsy is not routinely performed in RVCL. Although the diagnosis was established based on the characteristic histopathological pattern in conjunction with genetic findings, electron microscopy was performed to further characterize the microvascular alterations in this uncommon setting. Ultrastructural evaluation demonstrated endothelial electron-dense material and granular osmophilic deposits, supporting the presence of a primary microangiopathic process.

This case underscores several clinically relevant points. First, RVCL should be considered in the differential diagnosis of rim-enhancing white matter lesions, particularly in patients with a compatible family history or systemic features. Second, recognition of the characteristic histopathological pattern can prevent misdiagnosis as a primary brain tumor and avoid unnecessary oncologic treatment. Finally, correlation of radiological, pathological, and genetic findings is essential for establishing the diagnosis in rare hereditary small-vessel diseases.

## Conclusions

RVCL is a rare adult-onset small-vessel disease that can present with tumor-like white matter lesions, leading to frequent misdiagnosis as high-grade glioma or inflammatory demyelinating disease. This case underscores the importance of considering RVCL in patients with progressive neurological deterioration, suggestive family history, and atypical rim-enhancing lesions on MRI. The opportunity to perform histopathological and ultrastructural analysis provided direct evidence of the underlying vasculopathy, ischemic injury, and inflammatory changes characteristic of this disorder. Integrating clinical, radiologic, genetic, and pathological findings is essential to establish an accurate diagnosis and to avoid unnecessary oncologic interventions.
